# Veterinary Questions in the United States

**Published:** 1885-01

**Authors:** 


### EDITORIAL DEPARTMENT.

### VETERINARY QUESTIONS IN THE UNITED STATES.

With this number of the JOURNAL we begin a New Year, and earnestly hope that it may find each one of our readers more successful than any previous one has been. We shall spare no labor in our endeavors to still more markedly make our publication a Journal of Comparative Medicine, in fact, and must again reiterate our frequently repeated requests for assistance from the members of the profession. Without in any way desiring to pass any reflections upon other journals devoted to the interests of the Veterinary Profession in the United States, we feel that we can truly say that the JOURNAL OF COMPARATIVE MEDICINE is the only veterinary journal published in America which is exclusively devoted to the advancement of the profession, and which is absolutely free from any school or local entanglements. Wherever we find a school which deserves favorable comment, we are not afraid to give it any more than we are to condemn injurious actions on the part of any of them. In this regard we desire to speak a word about the schools. As the case now stands we know of but three schools on the American continent that are worthy of public patronage, and it should be the united endeavor of every honest veterinarian to support them to the best of his ability. These schools are the American Veterinary College of New York, the Veterinary Department of the University of Pennsylvania, and the college or school at Montreal. We are well aware that there are other schools in the country, but they have so much in question about them that we do not feel warranted in recommending them to students. We have before us a Chicago paper from which we have cut the following advertisements:

CHICAGO VETERINARY COLLEGE, Nos. 79 to 83 East 12th Street, Chicago, Ill. Established 1883. Chartered by the State of Illinois.
A. H. BAKER, V.S., *Principal*,
145 Michigan Avenue, Chicago, Ill.

---

MANGE IN DOGS. Use "Baker's Mange Cure." It kills the parasite every time. It is used by all the best breeders and fanciers in America. Price $1 per pint bottle. Regular discount to the trade. Agents wanted in every town. Address, A. H. BAKER, V.S.,
145 Michigan Avenue, Chicago, Ill.

The reader will observe that the principal of this so-called college is also the advertiser and proprietor of what every honest medical man looks upon as a Quack Medicine. The time has surely come in the United States when students must assume some responsibility as to the character of the schools from which they graduate. We go even so far as to look with disfavor upon the methods of all schools that advertise in sporting papers. With reference to the Philadelphia school, we have every reason to congratulate ourselves upon its foundation. Its principles are correct; it is gradually being well founded, but it will never be a veterinary school *par excellence* until every teacher is a veterinarian, and until it is rich enough to so pay them as to be able to forbid any teacher entering into competition with private practitioners by attending on patients outside of the school-hospital. The same objection is to be raised against the school of Montreal. It has been fully settled, in Europe, that M.D.s have not, in general, the ability to practically adapt the principles of medicine to the needs of veterinarians.

We cannot leave this subject without saying a word for a school which has been for so many years a great blessing to this country, and which has been the work of so many years of active devotion to the cause of our profession in the United States. Of course, we mean the American Veterinary College of New York. While this school has but a two years' course, it has still been a matter of surprise to us with what success its teachers have been enabled to educate a great number of unusually practical men. We have observed them carefully and sceptically, and must say that what we have seen of the alumni of this school have been exceptionally good routine practitioners, and what is more, that they have all had a certain respect for the true value of an advanced scientific education which, as the case has thus far been in our country, is an unnecessary qualification in the veterinarian.

We say "unnecessary qualifications!" Why? Because we have not a Veterinary Police Organization in any part of the country, though it is to be sincerely hoped that the nucleus has been laid in the present Bureau of Animal Industry.

The march of events is, however, assuming a very rapid, and at times, almost climaxical, advancement, which indicates that before long there will be an increased demand for men of exceptional ability by the various States. When this demand becomes active there will be a call for a school of a different and more advanced character, and we firmly believe that the labor and devotion of so many years will not have been spent in vain, and that the government of the United States will yet see the necessity of establishing a national institution for the development of comparative experimental and preventive medicine, which shall be second to none in the world. We believe in the possibility of such an institution. We know that the cost would be nothing to a country with over one hundred millions surplus revenue.

It is only necessary that these contagious animal diseases continue to make themselves more and more felt by the individual American; that each citizen become more and more educated in the intimate relation existing between many animal diseases and the public health; that the great intelligence of America is gradually brought to realize that the grand work of the study of causes of disease, especially those of an infectious nature, is dependent upon the development of comparative pathology, to have Congress realize that one of its duties is to establish such an institution.

The entire stock-raising, dairy, agricultural and hygienic interests, as well as the medical and veterinary professions, are blind to their duties, to themselves and the country unless they realize this point, and take active measures to bring it to the needs of their representatives in Congress.

The advocating of this idea, the endeavor to impress it upon the minds of the people, is as much a duty of the Commissioner of Agriculture and each State Board of Agriculture as is the endeavor to suppress existing diseases.

What we want more than anything else in the United States, except an honest government, is an institution for original research into the causes and nature of disease. It is a disgrace to so wealthy a country, a reflection on our practical common sense, that earnest men who love their country and humanity above all else, must go to Europe in order to fit themselves for such work. It is still more a disgrace to the country that there is no place for them to continue their labor on their return; but that instead of being valuable aids in the civilization and prosperity of the country, such men must be lost in the daily work of a routine practice.

We cheerfully admit being somewhat cranky upon this subject, but are willing to be found in the company of the Virchows, Pasteurs and Kochs of this world, whatever becomes of us in the next.

In our editorial capacity we have frequently requested our colleagues to send us reports of the occurrence of contagio-infectious diseases, and their observation as to their nature and causes of their extention; with what success our readers can best judge. The editor of the *American Veterinary Review* has also made a like request, with an equally favorable result. This will not do. A profession that is too indolent to help itself is unfit to exist.

The editor of the *Drover's Journal* seems to think that the whole purpose of the Bureau of Animal Industry is to make a raid upon the next Congress for funds. This person has of late been doing his utmost to bring our profession into disgrace by the publication of the most absurd libels about certain public officers. He asserts that there is not a single case of pleuro-pneumonia-contagiosa in the country, and even infers that there never has been. Happily we do not need to go far for our justification, as in the *National Live Stock Journal* —Chicago, September and October, 1884—we find its existence most strongly proved, even by autopsies. This journal does not appear to be any more favorable to the government veterinarians than the former.

The cases quoted by the *Drover's Journal* against former government officials have nothing to do with the present Bureau, unless it still retains those officials, of which at present we are uninformed. We have repeatedly referred to them, and equally consider them inexcusable; but we do not believe that there is no lung plague in the country any more than we do that the chief veterinarian of the United States would make a false statement, which both of these journals are doing their best to impress upon the minds of their readers. If it is the purpose of the Bureau to make a raid upon the public funds, its desires are very moderate in comparison to those which we have advocated for so many years, and which we shall continue to emphasize so long as we have power to wield a pen—

though we have no desire or expectation of being present at the annual divide, being what has been termed "uncontrolable, erratic, with some good points." One thing is sure, we would not falsify or keep silent over the discovery of a scientific fact if we saw the destruction of the whole animal industry before us. We discovered trichinæ in our pork to a very large degree. We know it is more so than any known pork. The effect of such a discovery is nothing to us. The fact exists. It is the duty of the government to find out the cause and prevent its action—not to suppress the fact. That this is the principle that rules our Chief Veterinarian may be seen from his own words:

"I can assure you that this department does not hesitate to diagnose a case of pleuro-pneumonia when it has sufficient evidence to base a diagnosis upon."

We shall never cease our work until we see a State Veterinarian in every State, and County Veterinarians wherever necessary, and every city with its municipal abattoirs and corps of competent inspectors.

No such officials should be appointed as they now are, but all should be previously subjected to an examination.

The present methods of slaughter-house inspection is a humbug. We have a right to know how many cases of disease occurs; the species and sex most frequently attacked, and the nature of the disease in every animal and organ. "So many pounds of meat condemned" means next to nothing.

Public hygiene in America is almost a farce in this regard as at present conducted.

We will again repeat what we have heretofore stated, that we still have the utmost confidence in the ability of the Chief Veterinarian of the Bureau of Animal Industry, though we do not believe in the possibility of his being able to extinguish pleuro-pneumonia from the West in six months, if there are six hundred cows there, as he is reported to have said he will do. Our Government Veterinarians have the same difficulties to overcome that are still troubling our English cousins. They have to contend with local and general rights. The people of this country have yet to learn that in the combatting of contagious animal diseases there is no such thing as local or State rights. This is a question over which our legislators will have to wrestle for many a day before they will come to any satisfactory or beneficial conclusion.

Pleuro-pneumonia can be stamped out of the United States in one year, but it will take the united endeavors of the owners of diseased animals, the public officials, the Government itself and the veterinary profession to do it. Once out, it can be kept out, but only by the employment of men of the most exact education and autocratic character by the Government in the place of the half-educated men many of its officials at present are, and too many have been. The only way to have good work done is to appoint men of this stamp, and have the laws so made that they shall retain their positions—unless neglectful of duty—no matter how much political opposition may cry out against the severity of their actions.

A writer in the *Live Stock Journal* for September, 1884, says: "Where the disease came from and how it was introduced into this State—Illinois—is a matter of no great importance." According to the same journal, the disease was introduced into Illinois by cows purchased in Ohio.

On the contrary, this is the most important question of all. We have quietly comforted ourselves that the disease is still in our sea-born States, yet with one jump we find it in Ohio, Illinois and Kentucky, and cannot say but what it will yet extend to the western plains.

With all charity, we must say that some one is to blame for this sudden extension of the disease beyond the Alleghanies, and that party is either the Government of the United States or that of the States.

The fault of the introduction and extension of contagious animal disease lies primarily with the Washington government, secondarily with the State governments.

We boldly assert that they cannot be controlled until the people demand the governments to give them a National Veterinary Police System of the character previously mentioned. State systems will not do. The laws must be National. The force National. State rights are a humbug when these questions come to be considered.

A National Veterinary Police Force is a national necessity.

The present schools cannot give us the men we want for such a force as they are now organized, hence we also require a national institute for the development of comparative and preventive medicine.

Pile up the statistics, veterinarians, of the occurrence of contagious diseases; Tuberculosis also in your districts; observe how they extend; report them to your journals; look upon the Bureau of Animal Industry as your friend, and allow no petty jealousies to lead you to lessen its influence. We cannot all be heads, but it takes just as good a man at the stern as it does on the lookout to keep the ship of State moving. B.

